# Application of Disinfectants for Environmental Control of a Lethal Amphibian Pathogen

**DOI:** 10.3390/jof7060406

**Published:** 2021-05-21

**Authors:** Leni Lammens, An Martel, Frank Pasmans

**Affiliations:** Wildlife Health Ghent, Department of Pathology, Bacteriology and Avian Diseases, Faculty of Veterinary Medicine, Ghent University, 9820 Merelbeke, Belgium; an.martel@ugent.be

**Keywords:** *Batrachochytrium dendrobatidis*, disinfection, amphibian, peracetic acid, mitigation

## Abstract

Chytridiomycosis is an emerging infectious disease threatening amphibian populations worldwide. While environmental disinfection is important in mitigating the disease, successful elimination of *Batrachochytrium dendrobatidis* (Bd) without excessively harming ecosystems is challenging. We selected peracetic acid (PAA) as the most potent of six commercially available products regarding their ability to inhibit growth of a highly virulent Bd strain. PAA killed Bd after 5 min of exposure to approximately 94.7 mg/L. We examined the toxicity of PAA against three invertebrate species and *Discoglossus pictus* tadpoles. 93% of invertebrates, but none of the tadpoles survived 5 min of exposure to 94.7 mg/L. Tadpoles showed no adverse effects after 5 min exposure to concentrations of approximately 37.9 mg/L or lower. Addition of PAA to aquatic microcosms decreased pH, while dissolved oxygen (DO) initially increased. Degradation of PAA reversed the pH drop, but caused a massive drop in DO, which could be remedied by aeration. As proof of concept, microcosms that were aerated and treated with 94.7 mg/L PAA sustained survival of tadpoles starting 48 h after treatment. Disinfecting aquatic environments using PAA could contribute to mitigating chytridiomycosis, while preserving at least some invertebrate diversity, but requires temporary removal of resident amphibians.

## 1. Introduction

For at least five decades, ecosystems have been experiencing biodiversity losses at alarming rates [1,2,3,4,5,6,7]. Amphibians are disproportionally affected, with an estimated 41% of known species listed as vulnerable, endangered or critically endangered according to the IUCN database [2,8]. Emerging infectious diseases have become an increasingly prevalent threat for biodiversity [9,10,11,12,13,14]. The disease chytridiomycosis has “caused the most prominent disease-driven loss of vertebrate diversity ever recorded” [9]. Causative agents of chytridiomycosis are the chytrid fungi *Batrachochytrium dendrobatidis* (Bd) [15] and *B. salamandrivorans* (Bsal) [16]. At least 501 amphibian species have been reported to suffer declines because of chytrid infection [9]. Curbing the impact of Bd and Bsal is therefore a major challenge in amphibian conservation efforts. Transmission without physical contact between hosts has been demonstrated in aquatic environments [17,18]. Therefore, environmental decontamination is considered key in mitigating amphibian chytrids [19,20], but the use of efficient yet aggressive disinfectants in nature is expected to have detrimental side effects. Therefore, screening alternative, effective products that are less toxic and quickly eliminated is of high priority for conservation efforts.

Several commercial, antimicrobial products have been developed for application in aquatic environments. Malachite green and formalin are examples of chemicals that are often applied in the aquaculture industry because of their low cost and broad parasiticidal and fungicidal capabilities [21,22], despite also being carcinogenic environmental contaminants [23,24,25]. Peracetic acid (PAA), on the other hand, is an example of a non-mutagenic chemical disinfectant that, upon rapid reaction with organic compounds, degrades into non-toxic compounds [26]. In light of this particular property, PAA is often used as an alternative for the aforementioned products in aquaculture for disinfection of fish eggs in hatcheries, among other things [27,28,29,30]. PAA is commercially available in acidic mixtures with water, hydrogen peroxide and acetic acid, described by the following equilibrium formula:

(1) $${\mathrm{CH}}_{3}\mathrm{COOH}+H_{2}O_{2}⇌{\mathrm{CH}}_{3}{\mathrm{CO}}_{3}H+H_{2}O$$

All of these compounds can be further broken down into water, oxygen and carbon dioxide. Although the antimicrobial effects of PAA have been the subject of several studies [31,32], there are no publications available on its usefulness as a disinfectant in amphibian habitats.

We screened six commercially available disinfectants and antifungal products for their effectiveness against growth of a highly virulent Bd strain. Of the most promising product, we also determined its Bd killing ability value. Then, we examined the toxicity of the selected compound against a phylogenetically wide range of aquatic animals. Finally, we studied the effects of the addition and degradation of the selected compound on an aquatic microcosm.

## 2. Materials and Methods

### 2.1. Bd Culture Maintenance

The highly virulent *Batrachochytrium dendrobatidis* strain JEL423 (isolated from *Phyllomedusa lemur* in Panama, 2004, and kindly provided by Dr. Joyce E. Longcore, University of Maine) used for the killing ability experiments was cultured in 250 or 550 mL cell culture flasks (CELLSTAR^®^ Greiner Bio-One GmbH, Germany) with filtered caps, filled with either 25 or 50 mL TGhL (16 g tryptone, 4 g hydrolyzed gelatine, 2 g lactose in 1 L of distilled water) broth, respectively. The culture grew in the dark at 20 °C. Zoospores were collected 1 day after TGhL broth changes on 4–5 days matured whole cultures, to ensure zoospore freshness.

### 2.2. Growth Inhibition of Bd by Six Commercial Antifungals

Motile spores of *Batrachochytrium dendrobatidis* JEL423 cultures were seeded in 24-well plates at a concentration of 2 × 10^5^ spores ml^−1^ in a 1:1 mixture of TGhL and distilled water. After 5 days of incubation at 20 °C, the wells were exposed to a twofold dilution series of one of six antifungal agents or disinfectants, selected for commercial availability: Blagdon pond anti-fungus and bacteria (Swell UK Ltd., Hyde, UK), F10^®^ SC (Health and Hygiene (Pty) Ltd., Randburg, South Africa), Morenicol FMC-50 (Colombo B.V., Ooltgensplaat, The Netherlands), povidone-iodine (Meda Pharma, Solna, Sweden), SERA pond omnisan (Sera GmbH, Heinsberg, Germany) or Wofasteril40 ^®^ (Kesla Pharma Wolfen GmbH, Bitterfeld-Wolfen, Germany). Dilutions were prepared using TGhL broth, in volume fractions at room temperature. Product quantities were later converted to mg/L using the density provided by the manufacturer. After application of the dilution series, the cultures were incubated at 20 °C and survival was visually determined 7 days later. The minimal inhibitory concentration (MIC) was per definition the lowest concentration at which none of the three replicates display spore activity or signs of growth.

### 2.3. Killing Ability Concentration Determination of Wofasteril40 against Bd

Motile spores of *Batrachochytrium dendrobatidis* JEL423 cultures were seeded in 24-well plates at a concentration of 2 × 10^5^ spores ml^−1^ in a 1:1 mixture of TGhL and distilled water. After 5 days of incubation at 20 °C, the wells were exposed per triplicate to a twofold dilution series of a peracetic acid (PAA) formulation (Wofasteril40 ^®^, Kesla Pharma Wolfen GmbH, Germany) in distilled water. Wofasteril40 was selected based on its efficiency to inhibit Bd growth. Dilutions were prepared in volume fractions at room temperature. Product quantities were later converted to mg/L using the density provided by the manufacturer. After a five minute treatment, cultures were rinsed with TGhL broth twice before adding a final volume of 1 mL TGhL. Subsequently, the cultures were incubated at 20 °C and survival was visually determined 7 days later. This procedure was repeated twice in order to obtain independent replicates.

This experiment was repeated twice using artificial pond water (0.2 g/L CaCl_2_, 0.1 g/L NaHCO_3_, 0.1 g/L sea salt) as PAA dilution medium for uniformity with the following experiments.

### 2.4. Acute Toxicity of Wofasteril40 against Tadpoles and Aquatic Invertebrates

Animals were exposed for five minutes to either artificial pond water or a 291.3 or 2913 mg/L dilution of Wofasteril40 in artificial pond water. Dilutions were prepared in volume fractions (1:4000 and 1:400) at room temperature. Product concentrations were later converted to mg/L using the density provided by the manufacturer. The concentrations were chosen based on the results of the killing ability assay described above. After rinsing twice with artificial pond water, animals were followed up for 7 days. We chose Mediterranean painted frog (*Discoglossus pictus*) tadpoles as a vertebrate representative, because it produces a typical anuran tadpole, representative of European herpetofauna, it can be procured from captive breeding and is a suitable model for future research purposes involving *Batrachochytrium dendrobatidis*: *Discoglossus pictus* frogs and tadpoles can become infected, but do not develop disease symptoms [33]. All experiments were approved by the ethical committee of Ghent University (EC2020/0032). Invertebrate species were: the waterlouse, *Asellus aquaticus* (phylum: Arthropoda), a pea mussel species, *Pisidium* sp. (phylum: Molluska, class: Bivalvia) and the Seminole rams-horn, *Planorbella duryi* (phylum: Molluska, class: Gastropoda). Invertebrate species were selected for the purpose of compiling a representative (simplified) invertebrate community of amphibian breeding ponds. The mollusks (*Planorbella duryi* and *Pisidium* mussels) were selected based on their occurrence in amphibian breeding ponds in Flanders and local availability. All mollusks were of genera that are often used as model organisms in toxicology research, e.g., [34,35,36,37,38]. Waterlice (*Asellus aquaticus*) are crustaceans and are equally typical occupants of amphibian breeding ponds. They are often used as animal model in ecotoxicity studies, e.g., [39,40]. There were five replicates per concentration/control for each species, except for *D. pictus*, where *n* = 9 for 291.3 mg/L and *n* = 1 for the 2913 mg/L Wofasteril40 dilution (this concentration was not further assessed in this species since acute mortality was observed). Additionally, *D. pictus* tadpoles were exposed to two more concentrations of Wofasteril40: 116.5 and 58.3 mg/L. Survival was determined daily, and dead animals were removed and stored in 10% formaldehyde. Animals were fed daily, with crumbled flakes for bottom-dwelling aquarium fish (Tetra Wafer Mix, Tetra^®^, Melle, Germany). They were kept in 3 mL of artificial pond water, in a 6-well plate, at temperatures between 15 and 17 °C with natural circadian rhythms. Water was changed every other day.

### 2.5. Exposure of Microcosms to Peracetic Acid

Ten 5 L plastic tanks (Tarrington House^®^) were filled with each 1.15 L pond water, acquired from a natural pond. Each tank also received a similar amount of pond bottom sludge from the same basin, suspended in 0.75 L pond water. To each tank, a piece of java moss (*Taxiphyllum barbieri*) of 2.00 ± 0.05 g (dipped dry with paper towels) and 15 cm of hornwort stalk (*Ceratophyllum demersum*) was added. The tanks were then left to rest for 24 h to allow the sludge to settle, with the lid partially opened to allow for circulation. After 24 h, each of the tanks was provided with a tea filter (Finum^®^, plastic and stainless steel) with a lid, attached with a rubber band, to facilitate inspection of the experimental animals. To each tea filter, two *A. aquaticus*, two *Planorbella duryi* and two *Pisidium* sp. mussels were added on top of a paraffin film bottom layer to avoid direct contact with the stainless-steel sieve bottom. All invertebrates and aquatic plants were collected from a freshwater pond in Belgium. After allowing the invertebrates to acclimatize for 3 h, 100 mL of a 5826 mg/L Wofasteril40 dilution in artificial pond water (0.2 g/L CaCl_2_, 0.1 g/L NaHCO_3_, 0.1 g/L sea salt) was added to five of the tanks, resulting in a final concentration of 291.3 mg/L. Dilutions were prepared in volume fractions at room temperature. Product quantities were later converted to mg/L using the density provided by the manufacturer. The other five tanks were treated with 100 mL artificial pond water, and served as negative controls. Tanks were incubated for 7 days at temperatures between 15 and 17 °C and with natural circadian rhythm. Thirty minutes after adding Wofasteril40, the peracetic acid concentration was determined using MQuant ^®^ colorimetric test strips with range 5–50 mg/L and 100–500 mg/L. *D. pictus* tadpoles were added to each of the tea filters 1 h after treatment in control and treated replicates (*n* = 1 per microcosm), and, for the treated replicates, again 4 (*n* = 1) and 7 days (*n* = 2) later. Tanks and animals were visually inspected daily. pH was followed up daily using either a Hanna Instruments HI-991003 portable pH meter or a VWR International^TM^ pH100. Deceased invertebrates were removed, and deceased tadpoles were preserved in 10% formalin.

Based on observations from the first microcosm exposure experiment, a second microcosm experiment was set up. Methods were identical, except for the animals added (*Asellus aquaticus* and pond snail *Radix* sp., due to temporary unavailability of *Planorbella duryi* and *Pisidium* sp.). Additionally, animals were added 48 h after adding peracetic acid. In addition, a novel treatment with five new replicates was included, which consisted of treatment with peracetic acid and subsequent aeration with an air pump. Dissolved oxygen and pH were measured daily using a WTW Multi 3430 DO (probe: WTW FDO^®^ 925) and pH (probe: WTW SensoLyt^®^ 900-P) meter, for 10 consecutive days after treatment.

For a final proof of concept, a third microcosm experiment was set up, with just five aerated replicates as described above, and no invertebrates. Forty-eight hours after the PAA treatment, 10 tadpoles were added to the tea filters in each tank. Tanks and tadpoles were visually inspected daily for 7 days. Dissolved oxygen and pH were measured daily using a WTW Multi 3430 DO (probe: WTW FDO^®^ 925) and pH (probe: WTW SensoLyt^®^ 900-P) meter.

### 2.6. Statistical Analysis and Graphical Visualisation

All graphs were made in R i386 v4.0.3 using the “ggplot2” and “tidyr” packages. Tables were designed using Microsoft Excel. Statistical analysis was performed using R i386 v4.0.3. Bonferroni correction for multiple testing was applied when appropriate. A power analysis was performed in R i386 v4.0.3 for the toxicity experiments, to assess the minimum number of animals required per treatment, in order to obtain a power of >0.9 (with a Bonferroni corrected significance level of 0.017 and assuming as extremes 100% survival in control and 100% mortality in the treated groups).

## 3. Results

### 3.1. Effectiveness of Commercial Products against Bd

The MIC-values of the six commercial antifungals are summarized in Table 1.

### 3.2. Killing Ability of Peracetic Acid against Bd

Bd JEL423 cultures survived at 145.6 mg/L (11.7–51.0 mg/L hydrogen peroxide, 36.4–58.3 mg/L PAA and 36.4–94.7 mg/L acetic acid), but not at 291.3 mg/L Wofasteril40 dilution (23.3–101.9 mg/L hydrogen peroxide, 72.8–116.5 mg/L PAA and 72.8–189.3 mg/L acetic acid) when exposed for 5 min. Whether the Wofasteril40 dilution was prepared with distilled water or artificial pond water did not influence this result.

### 3.3. Acute Toxicity of Peracetic Acid for Aquatic Animals

**Figure 1:**
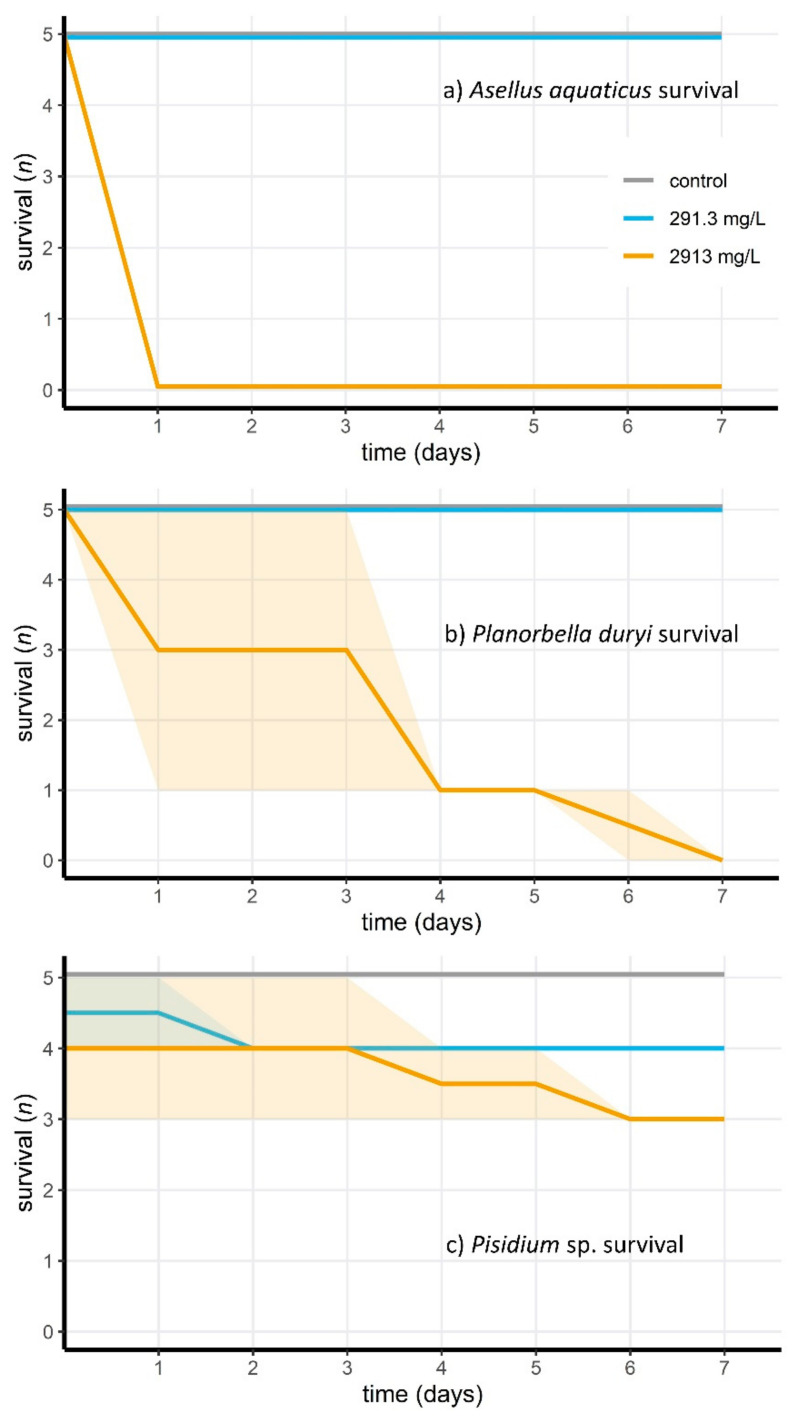
Survival of aquatic invertebrates ((**a**) *Asellus aquaticus*, (**b**) *Planorbella duryi* and (**c**) *Pisidium* sp.) after exposure to Wofasteril40 concentrations of 2913 mg/L (blue) and 291.3 mg/L (orange). Shaded areas indicate uncertainties about survival due to inactivity of the respective mollusk species.

**Figure 2:**
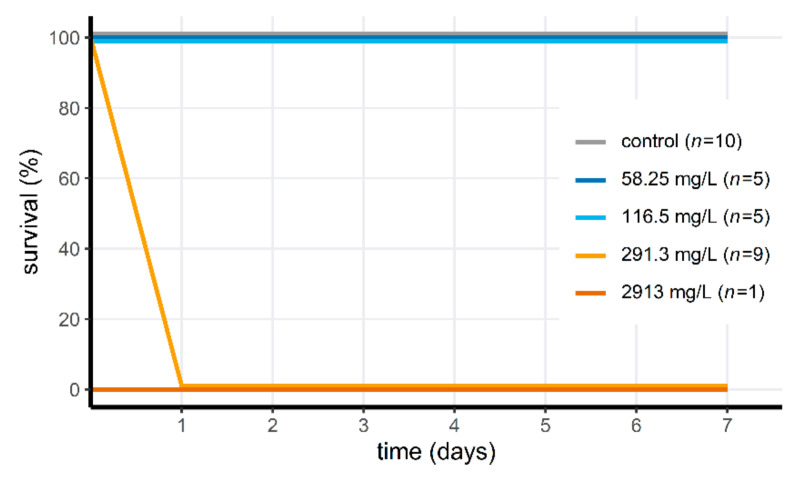
Survival of *Discoglossus pictus* larvae after exposure to peracetic acid concentrations of 58.25 mg/L (dark blue), 116.5 mg/L (light blue), 291.3 mg/L (light orange) and 2913 mg/L (dark orange). Notice the difference in number of animals (*n*) between the different concentrations.

Most of the aquatic invertebrates were tolerant to Wofasteril40 at concentrations of 291.3 mg/L (14/15 or 93% survival; 23.3–101.9 mg/L hydrogen peroxide, 72.8–116.5 mg/L PAA and 72.8–189.3 mg/L acetic acid), but not at 2913 mg/L (3/15, or 20% survival; see Figure 1a–c; 233.0–1019.4 mg/L hydrogen peroxide, 728.1–1165.0 mg/L PAA and 728.1–1893.1 mg/L acetic acid). Invertebrate survival differed significantly between low and high PAA concentration treatment groups (Fisher’s Exact test for Count data; *p* = 1.157 × 10^−4^). For the *D. pictus* tadpoles, Wofasteril40 was acutely toxic at high concentrations (Figure 2), but not at low concentrations. Survival differed significantly (Fisher’s Exact Test for Count data; *p* = 1.083 × 10^−5^) between groups exposed to concentrations of 116.5 mg/L (9.32–65.0 mg/L hydrogen peroxide, 29.1–46.6 mg/L PAA and 29.1–75.7 mg/L acetic acid) or lower (*n* = 10, mortality: 0%) and of 291.3 mg/L and higher (*n* = 10, mortality = 100%). Only one tadpole was tested at 2913 mg/L. This concentration was acutely toxic. The four other tadpoles from this treatment group were reassigned to the group exposed to 291.3 mg/L.

### 3.4. Peracetic Acid Concentrations Rapidly Decrease in the Microcosms

**Figure 3:**
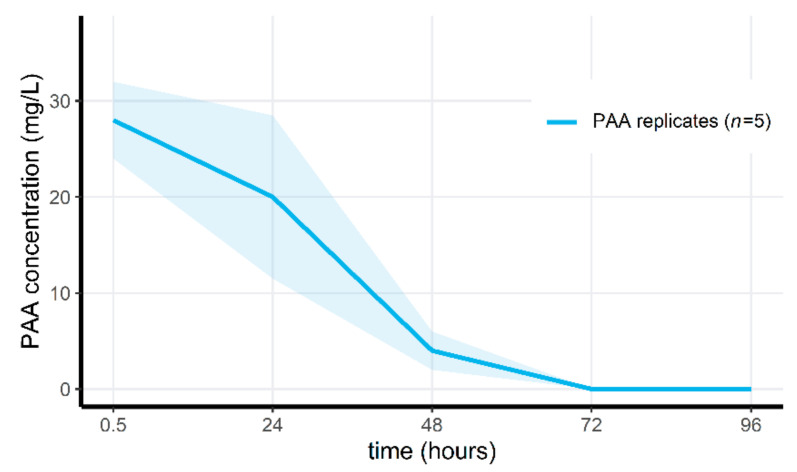
Mean measured PAA concentration through time in the PAA-treated replicates of the pilot microcosm experiment. Shaded areas indicate the mean ± standard deviations.

**Figure 4:**
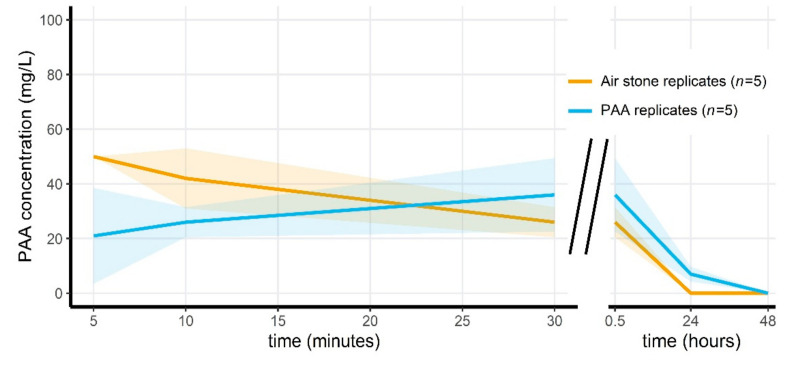
Mean measured peracetic acid concentration through time for the first 30 min ((**left**) in minutes) and for 24 and 48 h afterwards ((**right**) in hours) during the second microcosm exposure experiment. Shaded areas indicate the mean ± standard deviations. Measurements were taken at 5, 10 and 30 min, and every 24 h afterwards. Orange data is obtained from replicates that had an air stone and were treated with PAA, blue data is from the replicates that did not have an air stone and were treated with PAA.

Applying Wofasteril40 to the microcosms at 291.3 mg/L (23.3–101.9 mg/L hydrogen peroxide, 72.8–116.5 mg/L PAA and 72.8–189.3 mg/L acetic acid) resulted in the detection of 28 ± 4 mg/L peracetic acid after 30 min, for the pilot experiment. PAA concentrations decreased each day, until reaching below detectable concentrations after 72 h (see Figure 3). For the second microcosm experiment, PAA measurements are visualized in Figure 4. Here, PAA decreased to undetectable levels after 48 h in the non-aerated group, and after 24 h in the aerated treatment group. Similarly, PAA concentrations receded to below detectable levels after 24 h in the last microcosm experiment (see Supplementary Figure S3), which only consisted of aerated microcosms.

### 3.5. Adding PAA to Microcosms Results in a Temporary pH Drop

**Figure 5:**
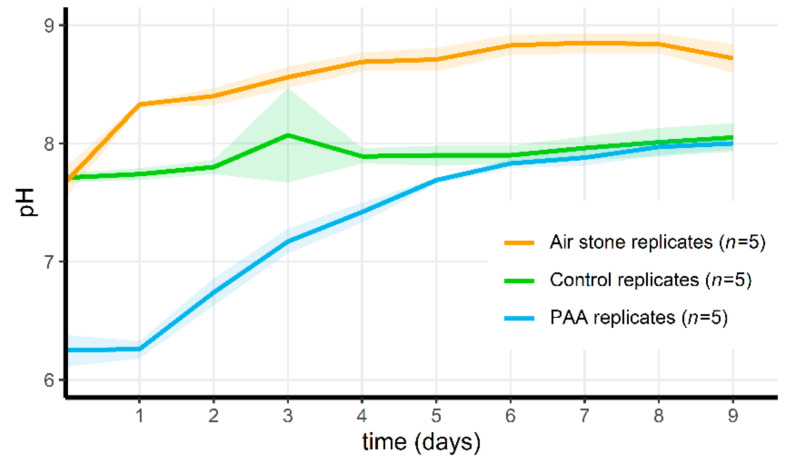
pH changes over time for the PAA-treated microcosms with air stones and PAA treatment (orange), the control microcosms (green) and the PAA-treated microcosms without air stones (blue) during the second microcosm exposure experiment. Shaded areas indicate mean ± standard deviation.

During both microcosm experiments, the pH of the pond water in the PAA-treated replicates was significantly different from the pH in control tanks for the first days after adding PAA (Wilcoxon test; *n* = 5 per treatment, *p* > 0.009 for the first two days in the first microcosm experiment, and *n* = 5 per treatment, *p* > 0.012 for the first 5 days in the second microcosm experiment). Subsequently, pH increased as the amount of PAA decreased in (Figure 5). Aeration of the microcosms prevented a pH drop after addition of PAA. The pH results from the pilot and third microcosm experiment can be found in Supplementary Figures S1 and S2.

### 3.6. PAA Treatment of Microcosms Results in a Drop of Oxygen Levels

All tadpoles (*n* = 5 on day 0, *n* = 5 on day 4 and *n* = 10 on day 7) survived until at least 2 h after being added to the tanks, but died within 72 h after introduction in the PAA-treated tanks during the first microcosm experiment, even after PAA levels had receded to undetectable levels. The invertebrates of the treated tanks died within the first 24 h after adding PAA and were not replaced. All control animals survived, except for one *Pisidium* mussel. Survival differed significantly between the control treatment and the PAA treatment for tadpoles (control tadpoles (n_total_ = 5) vs. tadpoles in treatment group (n_total_ = 20); Fisher’s Exact Test for Counting data; *p* = 1.882 × 10^−5^) and invertebrates (control (n_total_ = 30) vs. treated (n_total_ = 30); Fisher’s Exact Test for Counting data; *p* = 5.242 × 10^−16^).

**Figure 6:**
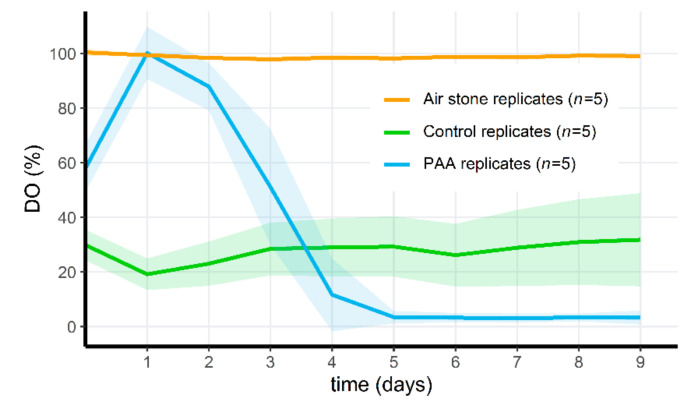
Mean dissolved oxygen (%) through time per treatment group for the second microcosm experiment. Shaded areas indicate mean ± standard deviation. Orange data is obtained from replicates that had an air stone and were treated with PAA, blue data is from the replicates that did not have an air stone and were treated with PAA, green data indicates results from the control replicates, that did not have air stones and were treated with artificial pond water.

**Figure 7:**
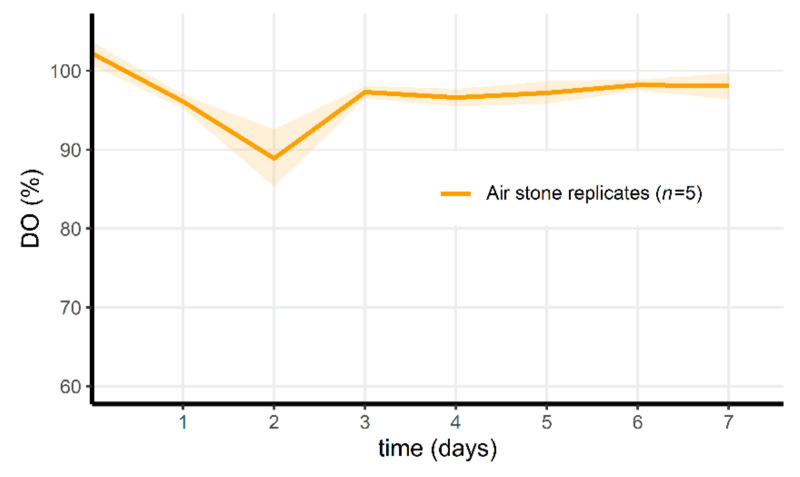
Mean dissolved oxygen (%) through time during the third microcosm exposure experiment. All microcosms were aerated and treated with PAA. Shaded areas indicate mean ± standard deviation. Notice the scale difference on the y-axis compared to Figure 6.

In a second microcosm experiment, we hypothesized that the mortality observed in this first experiment was initiated by a dramatic drop of dissolved oxygen (DO) concentrations (as seen in Figure 6). Initially, DO increased in the PAA microcosms compared to the control, but in the non-aerated replicates, the oxygen levels dropped quickly after the degradation of PAA. PAA differed significantly through time in this treatment group (Friedman test, *p* = 1.80 × 10^−5^). In the aerated PAA-treated group and the control group, oxygen parameters remained relatively stable, although the standard deviation of the control groups increased through time. Whereas the invertebrates in the non-aerated PAA treatment group died on the 6th day (*A. aquaticus*, *n* = 5) or appeared lethargic (*Radix* sp., *n* = 10), all of the animals in the aerated PAA-treated microcosms survived. In one control replicate, one *A. aquaticus* had also died on the 6th day. The dissolved oxygen in this particular control microcosm was never above 25%, and on the 7th day, reached a minimum of 7.3% (0.72 mg/L). In all other control microcosms, all animals survived, and oxygen levels were above 15% at all times. Survival did not differ between the three groups for *Radix* sp. (100%). Survival differed significantly for *A. aquaticus* between all groups (Fisher’s Exact 2 × 3 Test for Counting data; *p* = 5.994 × 10^−3^). Post hoc pairwise comparisons indicated a significant difference between the treated groups with and without aeration (Fisher’s Exact Test for Counting data; *p* = 7.937 × 10^−3^) but not between the control group and treated group without air stones (Fisher’s Exact Test for Counting data; *p* = 0.04762 (>0.017, Bonferroni-corrected *p*-value)) and between the aerated treatment group and the control group (Fisher’s Exact Test for Counting data; *p* = 1). The third microcosm setup served as a proof of concept. Tadpoles were introduced 48 h after PAA treatment, and all (*n* = 50) survived for 5 days in the aerated microcosms. A minor (approximately 10%) DO drop could be observed 2 days after application (Figure 7), as opposed to stable DO observations in the aerated treatment of the second microcosm experiment. On the 3rd day, DO had increased again and afterwards, oxygen concentrations remained stable.

## 4. Discussion

### 4.1. Effectiveness of Commercial Products against Bd

None of the commercial pond fish antifungal products were effective against Bd JEL423 at the concentrations recommended by the manufacturer: 22 mg/L vs. the effective dose of 100 mg/L dilution for Blagdon pond anti-fungus and bacterial, 40 mg/L vs. the effective dose of 160 mg/L dilution for Morenicol FMC-50 and 50 mg/L vs. the effective dose of 100 mg/L dilution for Sera pond omnisan. Furthermore, all of the pond antifungals contain malachite green as an active component, a known carcinogen [23] and slow-degrading ecological contaminant [24], arguing against their use in natural settings.

Of the disinfectants, povidone-iodine is the least effective, requiring 1250 mg/L dilutions to successfully cease Bd growth and spore activity. This is slightly higher than in a study from Berger et al. (2009) [41], where results of 3125 mg/L were obtained in a povidone-iodine MIC-experiment on Bd (strain: Tully N. dayi-1998-LB1) that lasted 4 days, but further followed similar methods. F10^®^ SC, a quaternary ammonium compound disinfectant, was the most effective product tested, requiring the lowest doses to inhibit growth and immobilize spores of Bd JEL423. The MIC we acquired (15.625 mg/L) was more than 6 times lower than the experimentally determined “no observable effect” concentrations (100 mg/L) for F10^®^ SC for various tadpole species during an exposure time of 15 min [42]. When exposed for 30 min, however, survival was limited to 70% for *Sclerophrys poweri* and 80% for *S. gutteralis*. Future research should be directed at investigating the killing ability of F10^®^ SC against Bd JEL423, as well as tadpole tolerance during longer exposure periods and degradability in (semi-)natural settings. It was decided to continue our investigation with the peracetic acid-based disinfectant Wofasteril40 ^®^ instead, because of the relatively low doses required to kill Bd, the rapid biodegradability of peracetic acid and its unknown toxicity to tadpoles.

### 4.2. Killing Ability of Peracetic Acid against Bd

Wofasteril40 is a mixture of PAA, hydrogen peroxide, acetic acid and water. It needs to be noted that these four components occur in a dynamic equilibrium with one another (see Equation (1)), which is influenced by environmental variables like temperature. To cope with this variability, we used the product range limits provided by the manufacturer for calculating actual concentration ranges of active products: 25–40 m% of PAA, 8–35 m% of hydrogen peroxide and 25–65 m% of acetic acid. The killing concentration we obtained for peracetic acid for Bd with a limited exposure time of 5 min was very similar to results obtained by Van Rooij et al. (2017) [43], where Bd JEL423 was tested against Kickstart^®^, another PAA-based disinfectant. In their study, 104 mg/L concentrations of PAA sufficed for killing off Bd cultures in 5 min, compared to our results of 72.8–116.5 mg/L. The killing ability concentration, however, was lower than the MIC value. This counterintuitive result can be explained by the difference in dilution medium in both tests; for the MIC-experiment, the product was diluted in TGhL in order to sustain the cultures during the long exposure period, while for the killing ability test, it was not necessary to dilute in TGhL broth. PAA reacts with the organic compounds in the broth, resulting in the need for higher initial concentrations to kill Bd than when using artificial pond water or distilled water as a dilution medium.

### 4.3. Peracetic Acid Concentrations That Kill Bd Are Acutely Toxic for Tadpoles

The concentration of PAA that quickly killed Bd was lethal for tadpoles after exposure for 5 min. This rules out the use of PAA as a treatment method for Bd-infected amphibians or its use in aquatic environments where tadpoles are present. The invertebrates were; however, rather tolerant to the killing ability concentration. Hence, it was decided to continue with the microcosm exposure experiment, to determine how long it would take for the microcosms to be viable again for tadpoles.

### 4.4. Disinfecting Aquatic Environments May Result in Drastic Ecosystem Changes

Adding peracetic acid (PAA) to the microcosms caused an immediate pH drop, as well as a sharp drop in dissolved oxygen (DO) levels. The latter explained mortality in the invertebrates and tadpoles. Initially, PAA increased DO, which can be explained by the breakdown of PAA into acetic acid and hydrogen peroxide, of which the latter ultimately degrades into H_2_0 and 0_2_, thus increasing DO. After this initial increase, the DO in the treated microcosms without air stones dropped to near negligible levels. A plausible explanation is the die-off of algae and other oxygen-producing micro-organisms, which then lead to oxygen depletion. Aeration reversed this oxygen drop and abolished the observed mortality in tadpoles and invertebrates. 

While the pH difference between the control and the PAA-treated microcosms was significant until 5 days post treatment this was unlikely to be the cause of tadpole mortality in the first experiment; Thabah et al. (2018) [44] reported partial survival of *Euphlyctis cyanophlyctis* and *Hyla annectans* for pH values of 3.5 and 4.5, respectively, when exposed for 48 h; Wijethunga et al. (2015) [45] reported partial survival of *Rhinella marina* at a pH value of 4 from egg deposit to metamorphosis; and Pierce and Montgomery (1989) [46] reported partial survival for *Xenopus laevis* and survival for *Bufo valliceps* and *B. woodhousei* at a pH value of 4 when exposed for 3 days. 

In conclusion, we demonstrate that, while several commercially available antifungals show promising activity against Bd in vitro, their use in the field is severely constrained by ecotoxicity issues. PAA showed marked anti-Bd activity, is quickly biodegradable and showed little toxicity for invertebrates tested. Its marked impact on pH and oxygen levels could be reversed by aeration. However, its impact on water chemistry and its acute toxicity for amphibian tadpoles preclude its use in vulnerable aquatic environments containing amphibian larval stages. Specifically, in settings (e.g., aquaria, artificial ponds or for disinfecting materials) where amphibians could be temporarily removed and aeration is feasible, PAA may prove useful in eliminating environmental contamination with Bd, which is deemed essential in disease control [19,47,48].

## Figures and Tables

**Table 1:** MIC-values (mg/L) of commercial products and their active components against Bd.

Product	
**Active Component**	**MIC (mg/L)**
**Blagdon^®^ pond anti-fungus and bacterial**	100
Malachite green oxalate	0.213
Acriflavine hydrochloride	0.437
**F10 ^®^ SC**	**15.625**
Alkyl (50% C14, 40% C12, 10% C16) dimethyl benzyl ammonium chloride	0.843
Poly (hexamethylene biguanide) hydrochloride	0.063
**Iso-bethadine ^®^**	**12,875**
Povidone-iodine	1250
**Morenicol^®^ FMC-50**	**160**
Malachite green	0.400
Methylene blue	0.080
Formalin	32.204
**Sera^®^ pond omnisan**	**100**
Malachite green oxalate	0.180
Formaldehyde	5.890
**Wofasteril40 ^®^**	**466**
Peracetic acid	116.5–186.4
Hydrogen peroxide	37.3–163.1
Acetic acid	116.5–302.9

## Data Availability

All measured data is made available in Supplementary Data S4.

## Supplementary Materials

The following are available online at https://www.mdpi.com/article/10.3390/jof7060406/s1, Figure S1: pH changes through time for the control microcosms (green) and the PAA-treated microcosms (blue) during the first microcosm exposure experiment. Shaded areas indicate mean ± standard deviation. Figure S2: pH changes through time for the third microcosm exposure experiment, only consisting of aerated microcosms treated with PAA. Shaded areas indicate mean ± standard deviation. Figure S3: PAA concentration for the third microcosm exposure experiment, only consisting of aerated microcosms after 5 and 30 min, and after 24 and 48 h. Shaded areas indicate mean ± standard deviation. Supplementary Data S4: an Excel file containing all measured data supporting reported results.
