# Supplementary figures and images for: Application of Disinfectants for Environmental Control of a Lethal Amphibian Pathogen
Source: J Fungi (Basel). 2021 May 21;7(6):406. doi: 10.3390/jof7060406 (PMC8224365; doi:10.3390/jof7060406)

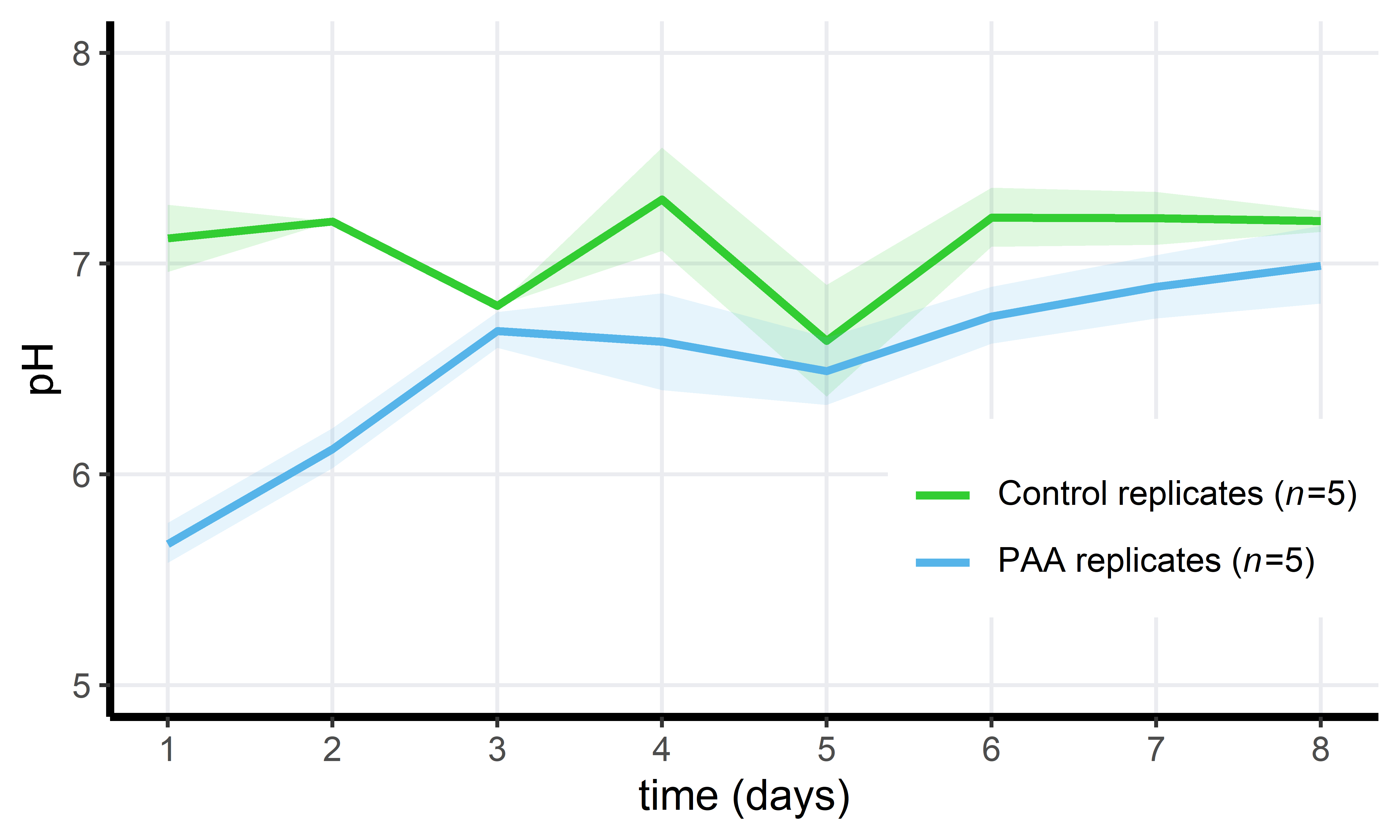

Supplement: Supplementary file 1 [file jof-07-00406-s001.zip › Lammens2021SI/Figure S1 pH first microcosm exp.tiff]

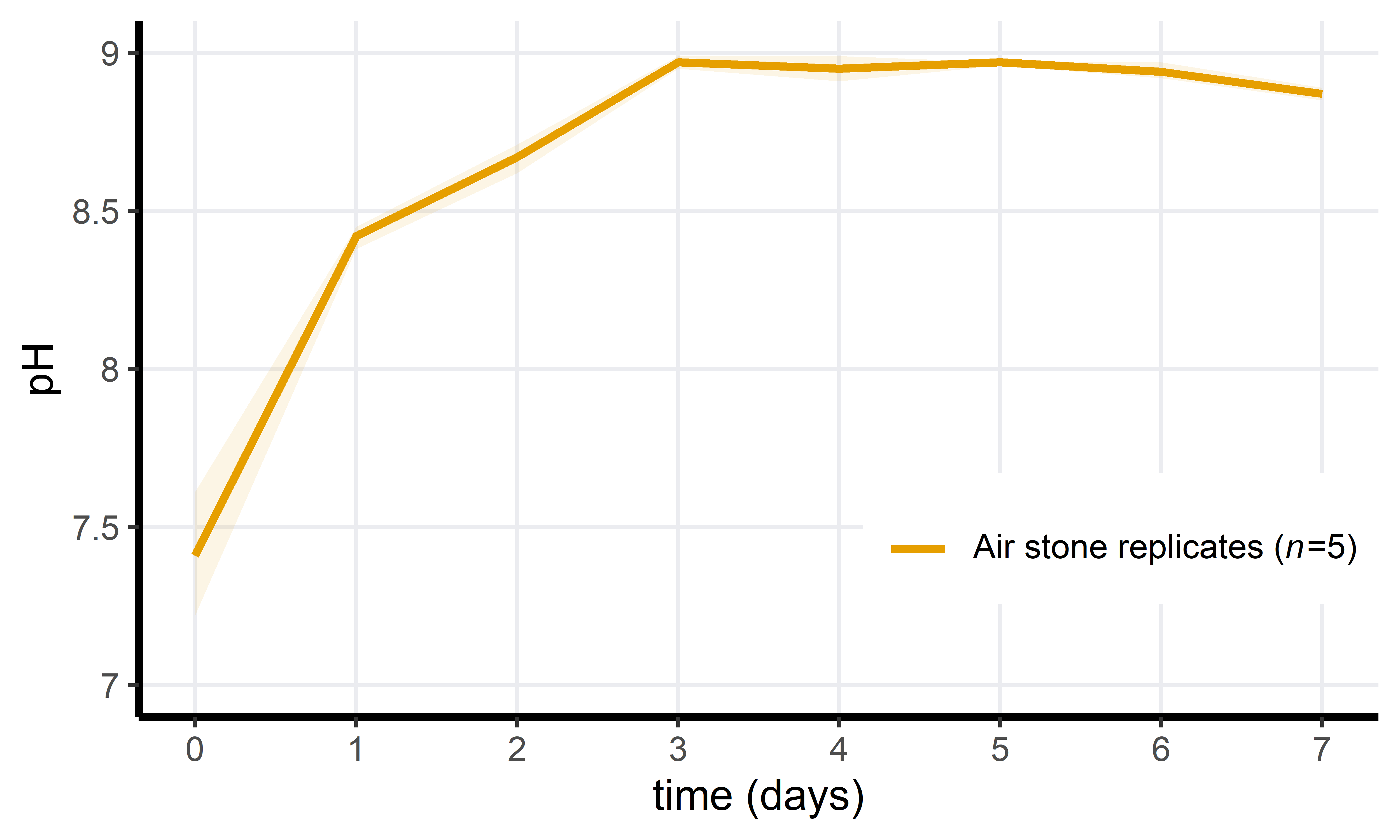

Supplement: Supplementary file 1 [file jof-07-00406-s001.zip › Lammens2021SI/Figure S2 pH third microcosm exp.tiff]

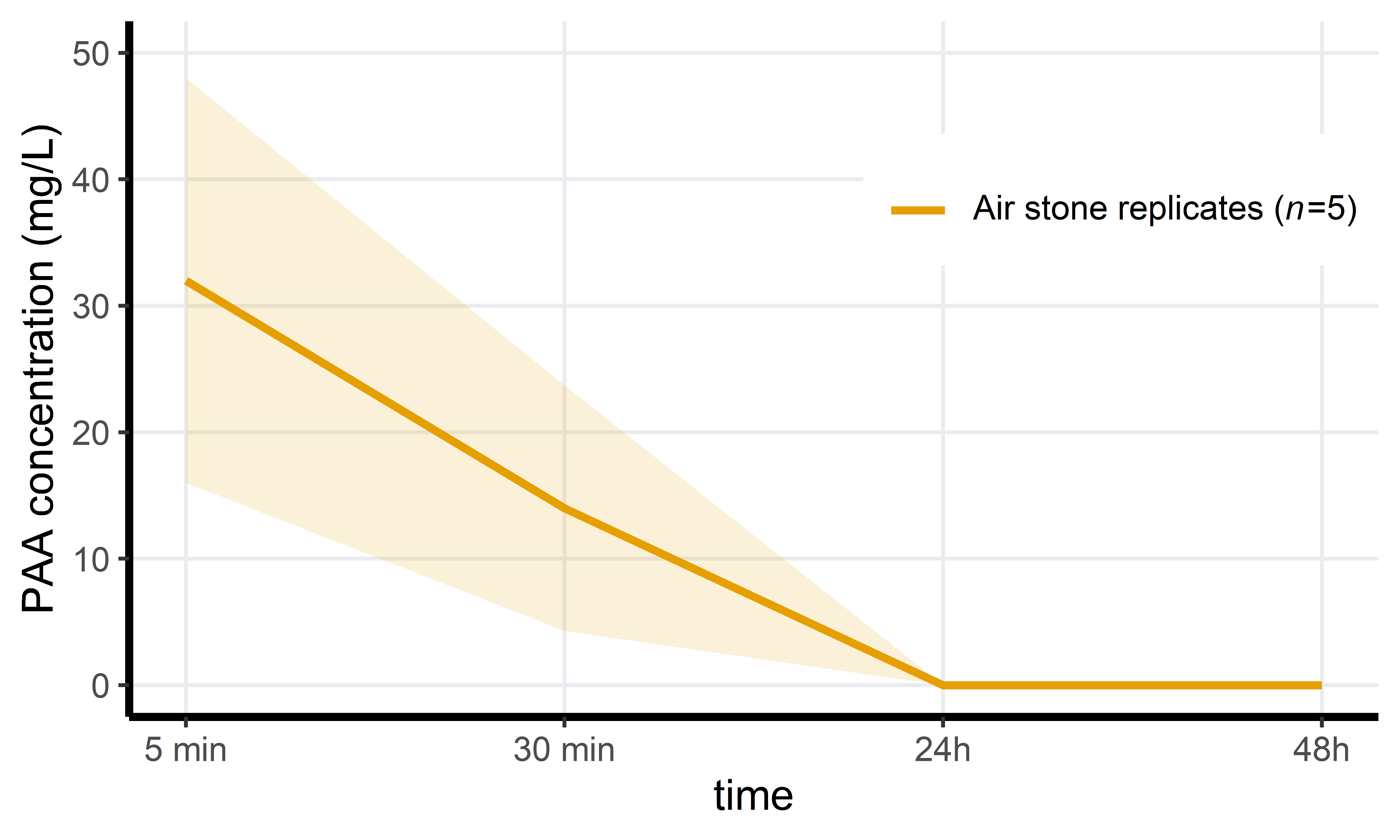

Supplement: Supplementary file 1 [file jof-07-00406-s001.zip › Lammens2021SI/Figure S3 PAA third microcosm exp.tiff]
